# Fathers’ and Mothers’ Early Mind-Mindedness Predicts Social Competence and Behavior Problems in Childhood

**DOI:** 10.1007/s10802-019-00537-2

**Published:** 2019-03-30

**Authors:** Cristina Colonnesi, Moniek A. J. Zeegers, Mirjana Majdandžić, Francisca J. A. van Steensel, Susan M. Bögels

**Affiliations:** 1 Research Institute of Child Development and Education, University of Amsterdam, Nieuwe Achtergracht 127, 1001 NG Amsterdam, The Netherlands; 2 Research Priority Area Yield, University of Amsterdam, Nieuwe Achtergracht 127, 1001 NG Amsterdam, The Netherlands

**Keywords:** Mind-mindedness, Fathers, Infancy, Developmental stability, Social competence, Behavior problems

## Abstract

Parental mind-mindedness, the parent’s propensity to treat the child as an intentional agent, has repeatedly shown to promote children’s development of social understanding and secure attachment. Less is known about whether the impact of maternal and paternal mind-mindedness extends to children’s social and behavior problems. We investigated the combined effect of mothers’ and fathers’ (*N* = 104) mind-mindedness at 4, 12, and 30 months on children’s social competence and externalizing and internalizing behavior problems at 4.5 years. Besides, we examined the stability, continuity, parental concordance, and inter-parental differences in the use of mind-related comments. Appropriate mind-mindedness (i.e., correct interpretations of the child’s mental states) and nonattuned mind-mindedness (i.e., misinterpretations of the child’s mental states) were observed during parent-child free-play interactions. Social competence, internalizing and externalizing behavior problems were assessed using both parents’ reports. Hierarchical multiple regression analyses showed that, at 30 months, infrequent use of appropriate mind-related comments of both parents predicted children’s externalizing problems, while their frequent use of nonattuned comments predicted children’s low social competence. Furthermore, mothers’ frequent use of nonattuned comments at 12 and 30 months and fathers’ nonattuned comments at 30 months predicted children’s externalizing behavior. The findings suggest that both parents’ low use of mind-related comments, and frequent misinterpretations of their child’s mind, may act as risk factors for later social and behavior problems of their child.

In modern societies, children start already at an early age to be involved in complex social contexts, such as preschool education, school, sports clubs, and friend groups. At this stage of life, teachers, friends, and other adults and children are increasingly taking part in children’s lives. Some children can fully participate and enjoy social interactions, whereas other children are socially less competent, or show externalizing or internalizing behaviors that prevent them from healthy interactions with others. Parent-child interactions in the first three years are likely to play a fundamental role in the child’s social-emotional development, as well as in reducing or preventing behavior problems (e.g., Carpendale and Lewis 2004; Lamb and Lewis 2010; Möller, Majdandžić, de Vente, & Bögels, 2013). Increasing attention has been drawn to a specific aspect of parent-child interaction, parental mind-mindedness, which refers to parents’ tendency to consider and to treat their children as individuals with an independent mind, rather than as entities with needs that must be met (Meins 1999, 2013).

Past research has demonstrated that maternal mind-mindedness in infancy is a predictor of child social understanding development, parent-child secure attachment (for a review, see McMahon and Bernier 2017; Zeegers et al. 2017), language development, and school readiness (Bernier et al. 2017; Meins et al. 2013b). Less is known about the predictive value of mind-mindedness on children’s social competence and behavior problems. Mind-mindedness is regarded as a manifestation of parents’ ability to “tune in” to what the child is thinking and feeling (Meins 2013). Doing so, parents directly enhance child trust in them, as well as self-regulation and socio-cognitive development (McMahon and Bernier 2017). For these reasons, parents’ mind-mindedness is expected to promote socio-emotional development and to reduce the risk of emotional, social, and behavior problems.

The concept mind-mindedness grew out of research aiming to study why infants become (in)securely attached to their caregivers (Meins 2013). The assessment approach of mind-mindedness during infancy entails free-play observations of caregiver-infant interaction, after which trained observers code how often the caregiver makes explicit appropriate and nonattuned mind-related comments toward the infant (Meins and Fernyhough 2015). Caregivers’ comments are mind-related when they refer to the infant’s mental states (e.g., cognitions, emotions, desires). Mind-related comments are, in turn, *appropriate* when they are correct reflections of the putative mental states of the infant (e.g., the mother saying “Oh darling, you are so sad” when the child is displaying sadness). In contrast, mind-related comments are *nonattuned* when there is no correspondence between the caregiver’s interpretation and the observable behavior of the infant (e.g., the mother saying “You like this turtle” when the child is actively engaged in playing with a different toy).

In general, about 6 to 8% of parents’ comments toward their baby during free-play situations are appropriate mind-related references. Nonattuned mind-related comments are produced far less frequently; they generally constitute around 1–2% of parents’ total amount of comments during free-play interactions (Arnott and Meins 2007; Kirk et al. 2015; Meins et al. 2003, 2012; Zeegers et al. 2017). In some studies it was reported that nonattuned comments occurred too infrequent to be included in the analyses (i.e., > 90% of the participants made no such comments at all); this was, for instance, the case in a low-risk sample (e.g., Bernier et al. 2017), or in professional caregivers (e.g., Colonnesi et al. 2017). The remaining comments of parents during interactions (± 90%) are not mind-related. These comments typically refer to the perception of the child, to what the child is saying or doing, to the parent’s mind or behavior, or to general conditions (e.g., “The sun is shining today”).

Appropriate and nonattuned mind-related comments are regarded as two orthogonal categories of comments (Meins 2013), as they are typically unrelated to each other (Meins et al. 2012). Moreover, meta-analytic data revealed that while mothers’ use of appropriate mind-related comments is positively associated with sensitive behavior (*r* = 0.30), mothers’ use of nonattuned comments is not related to (in)sensitive behavior (*r* = 0.13; Zeegers et al. 2017). For these reasons, appropriate and nonattuned mind-mindedness are commonly regarded as two distinct predictors of child outcomes (Meins et al. 2011). Parents with more optimal mind-mindedness often produce appropriate mind-related comments and seldom nonattuned mind-related comments.

Parental mind-mindedness is found to be stable in mothers during the first two years of life (Kirk et al. 2015; McMahon et al. 2016; Meins et al. 2011) and in the preschool years (Illingworth et al. 2016). No studies, however, have explored the temporal stability of mind-related comments in the transition from infancy to toddlerhood, which seems to be relevant for two reasons. First, stability from infancy to toddlerhood would confirm that mind-mindedness is a stable characteristic of the parent-child relationship (Kirk et al. 2015; Meins et al. 2011). Second, temporal stability would demonstrate the validity of measuring mind-related comments using an observation-based measure also beyond infancy, when children are able to communicate their thoughts and feelings verbally, and parents are therefore less challenged in interpreting children’s behaviors in terms of mental states. To date, four studies used observation-based measures of mind-mindedness with children of 2 to 10 years. In two studies mothers’ mind-mindedness was observed in parents of children aged between 19 months and 10 years (Fishburn 2017; Illingworth et al. 2016), using the coding system of Meins and Fernyhough (2015). In one study the Meins and Fernyhough coding system was used to observe interactions between professional caregivers and children of 3 years (Colonnesi et al. 2017). In another study, a different observational assessment method was used to assess mind-mindedness in mothers and fathers of 4-year-old children (Lundy 2013). Although these studies provide initial evidence of the possibility to measure mind-mindedness with observations after infancy, they did not test the temporal stability of observation-based measures of mind-mindedness from infancy till toddlerhood.

## Mothers’ and Fathers’ Mind-Mindedness

Research on the similarities and differences between mothers’ and fathers’ mind-mindedness is very limited. While mothers’ use of mind-related comments has been extensively investigated for almost 20 years, only a few studies explored both fathers’ and mothers’ spontaneous use of mind-related comments (Arnott and Meins 2007; Lundy 2003, 2013), providing some evidence of concordance and differences between partners.

Concordance refers to a positive association between fathers and mothers in the use of mind-related comments toward their child. Arnott and Meins (2007) reported a significant concordance between mothers’ and fathers’ appropriate mind-related comments at the age of 6 months (*r* = 0.44), while no concordance was found for nonattuned mind-related comments (*r* = 0.06). Similarly, Lundy (2013) found a significant concordance within mother-father couples in the use of mind-related comments with their 4-year-olds (*r* = 0.39). Concordance in mind-mindedness between parents suggests that parents influence each other and adopt similar modes of reading the inner states of their child (Arnott and Meins 2007).

Besides concordance, mothers and fathers could also differ from each other in how often and how accurate they comment on their infant’s mind. Arnott and Meins (2007) found no differences between maternal and paternal use of appropriate mind-related comments during the interactions with their 6-month-old infant. Fathers, however, displayed more nonattuned comments than mothers. Furthermore, fathers, but not mothers, who displayed more appropriate comments also produced more nonattuned comments. Lundy (2003) explored differences between mothers’ and fathers’ use of mind-related comments, using a similar coding system as Meins and Fernyhough (2015), in which only appropriate comments were coded. Also in this study, no differences were found between fathers and mothers in the overall number of appropriate mind-related comments toward their 6-month-old baby.

Due to limited empirical evidence and inconclusive findings, both the concordance and differences in the use of appropriate and nonattuned mind-mindedness between parents need to be further investigated. More importantly, since children typically have two parents, the joint effect of couples’ mind-mindedness should be investigated. A parent’s mind-mindedness may impact the child’s development independently of the other parent, as well as a parent may compensate or reinforce the impact of the other parent’s mind-mindedness.

## Parents’ Mind-Mindedness and Children’s Social Competence and Behavior Problems

An almost unexplored aspect of mind-mindedness is its predictive value on children’s development of social competence. Social competence refers to the ability to take the perspective of others, comfort and assist them, being collaborative and helpful, use negotiation, accept compromises, and be happy with one’s own accomplishments (Luteijn et al. 2000). Social competence has been found to be associated with emotion regulation abilities (Blair et al. 2015), attachment security (Groh et al. 2014), and Theory of mind (Devine et al. 2016), and all these three aspects have been found to be predicted by mothers’ mind-mindedness (e.g., Crucianelli et al. 2018; McMahon and Newey 2018 for emotion regulation; Meins et al. 2003 for attachment security; Kirk et al. 2015; Laranjo et al. 2014; Lundy 2013; Meins et al. 2013a, b for Theory of mind). One could, therefore, expect that parents’ mind-mindedness can stimulate children’s social competence by improving their early socio-emotional as well as their socio-cognitive development.

While a high level of mind-mindedness can stimulate a healthy social development in children, a low level of mind-mindedness can be a risk factor for children’s behavior problems (Meins et al. 2013a, b). Only a few recent studies have explored the association between mothers’ use of mind-related comments and children’s behavior problems. Meins et al. (2013a, b) report a negative association between mothers’ use of appropriate mind-related comments toward their 8-month-old infant and externalizing and internalizing behaviors at 4 years, but only in families with low SES. Camisasca et al. (2018) also found significant concurrent negative associations between maternal appropriate mind-mindedness and children’s internalizing and externalizing problems at 17 months. In contrast, Centifanti et al. (2016) found no association between maternal use of appropriate mind-related comments at 8 months and children’s behavior problems at 5 years. In the study of Easterbrooks et al. (2017) no direct relation was found between teen-mothers’ use of appropriate mind-related comments at 12 months and children’s behavior problems one year later. Taken together, these studies partially support the idea of an association between the use of appropriate mind-related comments and children’s later behavior problems.

Two crucial aspects of mind-mindedness need further attention. First, the relation between nonattuned mind-mindedness and behavior problems is yet unexplored, despite its acknowledged negative impact on children’s development of secure attachment (Meins et al. 2012; Zeegers et al. 2017), and early emotion regulation (Crucianelli et al. 2018; McMahon and Newey 2018; Zeegers et al. 2018). Parents’ continuous misunderstanding of the child’s inner states may hinder the quality of the relationship and the child’s subsequent socio-emotional development. Second, since infants are usually exposed to both mothers’ and fathers’ mind-mindedness from birth, the impact of both parents, as well as the possible interaction between parents, should be taken into account when exploring the impact of mind-mindedness on children’s development of social competence and behavior problems.

## The Present Study

The present study investigated to what extent mothers’ and fathers’ use of appropriate and nonattuned mind-related comments in early infancy (4 months), late infancy (12 months) and toddlerhood (30 months), as well as the interaction between mothers’ and fathers’ mind-mindedness, predict children’s social competence and behavior problems at 4.5 years. Before testing the main hypotheses, we tested the stability (rank order stability over time) and continuity (mean level stability across time) of parents’ mind-mindedness in the first three years, as well as fathers’ and mothers’ concordance (correlation) and differences (in levels) in the use of appropriate and nonattuned mind-mindedness.

We expected both mothers’ and fathers’ appropriate mind-mindedness to predict higher levels of social competence and fewer behavior problems at 4.5 years, and both mothers’ and fathers’ nonattuned mind-mindedness to predict children’s lower levels of social competence and more behavior problems at 4.5 years. Furthermore, the interaction between fathers’ and mothers’ mind-mindedness was investigated to test whether better social competence and fewer behavior problems were predicted by high levels of appropriate and low levels of nonattuned mind-mindedness in one or both parents. These analyses provided insight into how the combination of mothers’ and fathers’ levels of mind-mindedness predicts children’s social competence and behavior problems.

## Method

### Participants

The sample consisted of 104 first-born children (55 girls) and their parents participating in a longitudinal study on the antecedents of anxiety from infancy to middle childhood (de Vente et al. 2011). The parents were recruited during the pregnancy of their first child through obstetrician offices, advertisements in magazines, at pregnancy courses, and baby shops. One-hundred-and-one parents reported being married or having a cohabitation agreement while information about three couples was missing. The average length of their relationship was 6.23 years (*SD* = 3.60). The data of the present study are based on four measurements: 4 (4 m), 12 (12 m), 30 (30 m) months, and 4.5 years (4.5 y). The original sample consisted of 151 parent couples who started participating in the study during pregnancy (i.e., questionnaires). Fourteen families dropped out before T1 because they found participation with their young baby too time-consuming. Sample size dropped to 120 families at T4. The main reasons, as reported by the participants, were finding participation too time-consuming or having moved out of the area. Inclusion criteria for the present study were: availability of the data at 4.5 y of at least one of the two parents (dependent variables), and availability of mind-mindedness data for at least two of the three measurement times of at least one of the two parents (4 m, 12 m, and 30 m). We performed t-tests to examine significant differences between the sample with full data and the excluded sample within the same measurement on the levels of appropriate and non-attuned mind-mindedness. We applied a false discovery rate of 0.05 (Benjamini and Hochberg 1995) to correct for the vast number of comparisons (12). The analyses yielded no differences between the full and excluded sample.

The mean age of the children (in months) was: 3.73 (*SD* = 0.42) at 4 m, 11.87 (*SD* = 0.76) at 12 m, 29.59 (*SD* = 0.60) at 30 m, and 53.41 (*SD* = 0.63) at 4.5 y. About 93% of the mothers and 96% of the fathers were born in the Netherlands, and all parents spoke fluent Dutch or English. Mothers’ and fathers’ average age at 4 m was 31.37 years (*SD =* 4.34) and 34.10 years (*SD* = 4.90), respectively. The majority of parents had a high educational level (postsecondary school education or university): 81.2% of the mothers and 65.2% of the fathers; 15.8% of the mothers and 30.8% of the fathers had a medium level of education (secondary school); and 3.0% of the mothers and 6.7% of the fathers had a low level of education (basic school to lower secondary school).

### Procedure

Each mother and father independently visited the Family lab of the Research Institute of Child Development and Education (UvA) with their child at 4 m, 12 m, and 30 m. During each visit, we conducted a free-play task for the assessment of mind-mindedness. At 4.5 y both parents were asked to fill out questionnaires to assess their child’s social competence and behavior problems.

### Measures

#### Mind-Mindedness

At 4 m, mothers’ and fathers’ mind-mindedness was assessed during a 5-min free-play session, 2.5 min without toys and 2.5 min with a box of age-appropriate toys. Parents were instructed to play with their child as if they were at home. At the 12 m and 30 m measurements, parental mind-mindedness was assessed similarly, but the free play session lasted for 10 min and began with the parent and child seated on a play mat in the center of the room.

Each comment of the parent was transcribed and coded by trained observers by watching the filmed interaction (training intraclass correlation coefficient (ICC) > 0.80) using Meins and Fernyhough’s guidelines (2015). First, each comment of the parent was classified as either directed at the child’s mental state or not (i.e., mind-related or not mind-related). Mind-related comments were comments that referred to the child’s emotions (e.g., “You are frustrated”), cognitions (e.g., “You remembered how to make this puzzle”), desires/preferences (“You like to play with the ball”), epistemic states (“You are teasing me”), or comments that were clearly dialogue intended to be spoken by the infant (e.g., the mother says: “That’s a teddy bear, Mummy”). Second, each mind-related comment was coded as appropriate if: (a) the trained coder agreed with the parent’s reading of the infant’s internal state, and/or (b) the internal state comment linked the infant’s current activity with similar events in the past or future, or (c) the parent verbalized what the child might say if he/she could speak. Comments were classified as nonattuned when the coder believed: (a) the parent misread the internal state of the child, or (b) the comment referred to a past or future event that had no apparent relation to the infant’s current activity. After categorizing each comment, percentages of mind-mindedness were calculated ((*N* of appropriate or nonattuned mind-related comments/total number of comments)*100; Meins and Fernyhough 2015). These percentage scores of appropriate and nonattuned mind-mindedness were used in the analyses.

Inter-rater reliability was assessed on 110 out of 584 transcripts (19%). First, we assessed the inter-rater agreement on the number of mind-related comments using ICCs (two-way random effects model with an absolute agreement definition). Inter-rater agreement at 4 m was ICC_father_ = 0.75, ICC_mother_ = 0.88; at 12 m ICC_father_ = 0.84, ICC_mother_ = 0.84; and at 30 m ICC_father_ = 0.86, ICC_mother_ = 0.86. Next, we assessed the inter-rater agreement on appropriate and nonattuned comments by calculating Cohen’s Kappa. Interrater agreement at 4 m was к_father_ = 0.82; к_mother_ = 0.85, at 12 m к_father_ = 0.92; к_mother_ = 0.88, and at 30 m: к_mother_ = 0.79; к_father_ = 0.82. Disagreements were resolved through discussion.

Parents’ use of nonattuned mind-related comments was sufficiently frequent to be included in the analyses: the number of mothers who produced at least one nonattuned mind-related comment was 27 at 4 m, 31 at 12 m, and 29 at 30 m, while the number of fathers who produced at least one nonattuned mind-related comment was 34 at 4 m, 47 at 12 m, and 27 at 30 m.

#### Social Competence and Behavior Evaluation-30 (SCBE-30) (4.5 y)

Fathers’ and mother’s ratings on the Social Competence and Behavior Evaluation-30 for preschoolers (SCBE-30; LaFreniere and Dumas 1996) were used to assess children’s social competence and behavior problems*.* The SCBE-30 is a 30-item questionnaire designed to assess child emotional and behavior problems and social skills (Likert scale from 1 (never) to 4 (always)). The questionnaire includes three subscales: social competence (i.e., positive qualities in a child’s social adaptation), externalizing behavior (i.e., angry, aggressive, egotistical, oppositional), and internalizing behavior (i.e., depressive, anxious, isolated, dependent). A previous validation study has shown good internal consistency and construct validity (Kotler and McMahon 2002). In the present study, reliability and correlations between parents were: α _mother_ = 0.74; α _father_ = 0.75, *r*(104) = 0.36, *p* < 0.001 for social competence; α mother = 0.78; α father = 0.78, *r*(104) = 0.34, *p* < 0.001 for externalizing behavior; and α _mother_ = 0.82; α _father_ = 0.74, *r*(104) = 0.40, *p* < 0.001 for internalizing behavior.

#### Children Social Behavior Questionnaire (CSBQ) (4.5 y)

Child social behavior was also measured using parents’ ratings on the Children Social Behavior Questionnaire (CSBQ*;* Luteijn et al. 2000; Luteijn et al. 2002). The CSBQ assesses a broad range of behavior and emotional problems that are typical, but not necessarily specific, of children with a milder form of PDD (Pervasive Developmental Disorder). The parent report includes 49 items that are rated on a three-point scale (0 = does not apply; 1 = sometimes or somewhat applies; 2 = clearly or often applies). The questionnaire comprises six subscales (behaviors not appropriate to the situation, withdrawal, orientation problems, difficulties understanding social information, stereotyped behaviors, and fear of and resistance to change), and a total score of social difficulties. For the present study, we used the total score as a general measure of children’s social difficulties. Psychometric qualities of the questionnaire were reported to be good (Luteijn et al. 2000, 2002). Reliability in the present study was high: α _mother_ = 0.91; α _father_ = 0.91, and the correlation between parents was *r*(97) = 0.26, *p* = 0.009. In the present study, 31% (*n* = 32) of the children scored above the clinical cut-off of >23 (95th percentile; Luman et al. 2009) for social behavior problems.

### Statistical Approach

#### Study Variables

Mothers’ and fathers’ percentages of appropriate and nonattuned mind-related comments (at 4 m, 12 m, 30 m) were used as measures of their level of mind-mindedness. Children’s social competence index (at 4.5 y) was created by combining (after z transformations) the scores of the scale social competence of the SCBE-30 with the reversed score of the CSBQ, *r*(103) = 0.51, *p* = 0.001. The scales of externalizing behavior and internalizing behavior of the SCBE-30 were used as indexes of children’s behavior problems. Since mothers’ and fathers’ scores of social competence, externalizing behavior and internalizing behavior were positively and significantly associated (see previous two paragraphs), we averaged them into composite scores to generate robust measures of children’s behavior and to avoid multiple comparisons. Moreover, the results obtained with the composite measures were consistent with the results obtained with the single reporter measures.

#### Missing Value Analysis and Data Screening

Of the 104 participants, 4 m data on mind-mindedness was available for 96 mothers and 95 fathers. The 12 m data on mind-mindedness was available for 97 mothers and 98 fathers, and 30 m data on mind-mindedness was available for 100 mothers and 98 fathers. The scores on mind-mindedness were missing due to: no lab visit at one of the time points (*n* = 14), parents speaking foreign languages during the interaction (*n* = 6), and technical problems (*n* = 20). Because only 4.75% of the data had missing values, only predictors had missing values, and values were missing completely at random (Little MCAR test was not significant, χ^2^(151) = 171.82, *p* = 0.118), missing values were estimated by using the SPSS Expectation Maximization (EM) procedure (Graham 2009).

Fathers’ and mothers’ percentages of appropriate mind-related comments were normally distributed (values of skewness/*SE*_skew_ and kurtosis/*SE*_kurt_ were between −1.96 and + 1.96), while the percentages of nonattuned mind-related comments were positively skewed. Children’s scores on social competence and internalizing behavior were normally distributed. We detected one outlier (+ 3 *SD*) for externalizing behavior. The value was winsorized by modifying its value to the closest observed values. Afterward, children’s score on externalizing behavior was normally distributed.

#### Data Analyses

In order to investigate the temporal stability of mothers’ and fathers’ use of appropriate and nonattuned mind-related comments from 4 m to 30 m, four autoregressive models using structural equation modeling were conducted (Bollen and Curran 2004). The Lavaan package was used in the statistical software program R to analyze the model. The concordance between parents was investigated using Pearson’s correlations. Temporal continuity (mean level stability) of appropriate and nonattuned mind-mindedness over time, and differences between fathers and mothers at different ages, we tested using repeated measures ANOVAs on parental mind-related comments with parent (mother and father) and time (4 m, 12 m, and 30 m) as within-subjects variables.

Next, three-steps hierarchical linear regression analyses were performed to investigate whether mothers’ and fathers’ use of appropriate and nonattuned mind-related comments at 4 m, 12 m, and 30 m predict children’s social competence, and externalizing and internalizing behaviors. Following a chronological order, fathers’ and mothers’ mind-related comments at 4 m were entered at step 1, the same variables at 12 m were entered at step 2, and at 30 m at step 3. An interaction term (mother x father) was added to all three steps to test whether one parent’s mind-mindedness compensates for or ameliorates the effect of the other parent’s mind-mindedness. A significant regression model indicated at which age parents’ mind-mindedness significantly contributed to the variance in children’s competence and behavior problems. Significant interactions were further examined using the macro PROCESS 3.1 (Hayes 2013), using the Pick-a-point technique to probe the effect. The Pick-a-point technique allowed us to ascertain whether one parent’s (i.e., mother) use of mind-related comments predicted children’s social competence and behavior problems when the other parent’s (i.e., father) use of mind-related comments was low (1 *SD* below the mean), medium number (mean), and high (1 *SD* above the mean).

Preliminary tests on the assumption for the autoregressive models and multiple regressions indicated that the collinearity statistics were all within acceptable limits. Since the percentages of nonattuned mind-mindedness were not normally distributed, log-transformation of the data was applied prior to the autoregressive analyses and multiple regressions only (Tabachnick and Fidell 2013, pp. 86–88). After the transformation, residual and scatter plots showed that the assumptions of normality, linearity, and homoscedasticity were all satisfied. The Mahalanobis distance scores indicated no multivariate outliers.

## Results

### Preliminary Results

Table 1 shows the descriptive statistics and zero-order correlations of the study variables. Preliminary analyses on the impact of child sex, parents’ age, level of mothers’ education, and length of the relationship revealed no significant effects on the study variables, range *r* from −0.17 to 0.15, *p* from 0.092 to 0.132. A significant correlation was found between fathers’ education and children’s social competence, *r*(103) = 0.26, *p* = 0.008. Girls were rated more socially competent than boys (*M*_girls_ = 3.04, *SD* = 0.35; *M*_boys_ = 2.85, *SD* = 0.33), *t*(102) = 2.96, *p* = 0.004. Regression analyses on social competence were, therefore, conducted with and without controlling for child’s sex and fathers’ education (at step 1), leading to similar results. The results are presented without controlling for child’s sex and fathers’ education.

**Table 1:** Descriptive statistics and correlations of the study variables

Variables	*M*	*SD*	*Range*	AMRC 12 m	AMRC 30 m	NAMRC 4 m	NAMRC 12 m	NAMRC 30 m	SocComp 4.5 y	Extern 4.5 y	Intern 4.5 y
Mother											
AMRC 4 m	7.49	4.90	0–20.51	0.36**	0.08	−0.03	0.06	−0.16	−0.00	−0.08	0.04
AMRC 12 m	5.05	3.16	0–14.71	–	0.29**	−0.01	−0.18	−0.11	−0.06	−0.01	−0.11
AMRC 30 m	3.70	2.25	0–10.28		–	−0.01	−0.02	0.00	−0.12	−0.03	0.05
NAMRC 4 m	0.66	1.17	0–5.00			–	−0.04	0.15	−0.04	0.09	−0.00
NAMRC 12 m	0.33	0.53	0–2.33				–	0.04	−0.24*	0.20*	0.25*
NAMRC 30 m	0.26	0.46	0–2.17					–	−0.19	0.08	0.08
Father											
AMRC 4 m	7.99	5.90	0–28.00	0.33*	0.11	0.08	0.11	0.00	0.14	−0.20*	−0.02
AMRC 12 m	5.59	3.40	0–14.63	–	0.11	0.20*	0.19	−0.04	0.12	−0.15	0.07
AMRC 30 m	4.06	2.14	0–9.40		–	0.14	0.06	0.20*	0.17	−0.18	0.23*
NAMRC 4 m	1.43	2.40	0–11.94			–	0.48**	0.02	−0.05	0.09	−0.10
NAMRC 12 m	0.92	1.28	0–6.19				–	0.22*	−0.20*	0.15	0.01
NAMRC 30 m	0.34	0.59	0–2.15					–	−0.24*	0.21*	−0.04
Child											
SocComp 4.5 y									–	−0.54*	−0.26**
SC	2.95	0.35	1.80–3.70								
SP	19.85	9.58	1.50–50.00								
Extern 4.5 y	1.58	0.29	1.50–2.50							–	0.12
Intern 4.5 y	1.49	0.29	1.00–2.30								–

AMRC = Percentage of appropriate mind-related comments; NAMRC = Percentage of nonattuned mind-related comments; SocComp = Social Competence (SC = Social competence in SCBE-30; SP (not-reversed) = in CSBQ); Intern = Internalizing behavior. * *p* < 0.05; ** *p* < 0.01

### Mothers’ and Fathers’ Mind-Mindedness

#### Temporal Stability

**Fig. 1:**
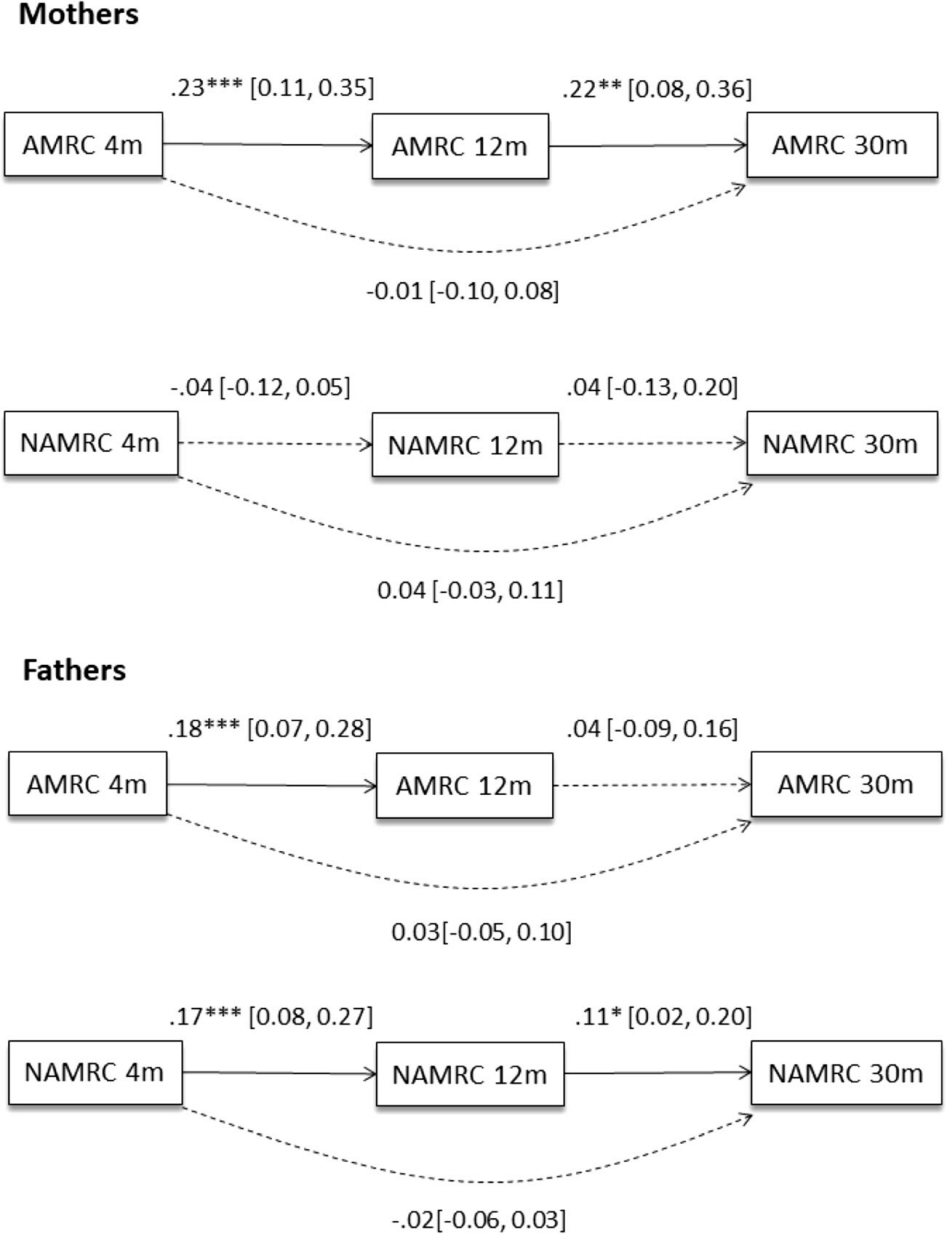
Autoregressive Model Testing Mother and Father Stability in the use of appropriate and nonattuned mind-related comments at 4 m (4 months), 12 m (12 months), and 30 m (30 months). *Note*. AMRC = Appropriate Mind-related comments; NAMRC = Nonattuned mind-related comments. *p < 0.05; **p < 0.01, *** *p* < 0.001

Figure 1 presents the autoregressive models showing the temporal stability of parents’ mind-related comments over time. Mothers’ use of appropriate mind-related comments was stable from 4 m to 12 m, as well as from 12 m to 30 m. Mothers’ nonattuned comments, on the contrary, were unstable over time. Fathers’ appropriate mind-related comments were stable from 4 m to 12 m, but not from 12 m to 30 m, and their nonattuned comments were stable from 4 m to 12 m, and from 12 m to 30 m.

#### Concordance between Parents

Couple concordance for appropriate mind related comments was not significant at 4 m, *r*(103) = 0.05, *p* = 0.631, significant at 12 m, *r*(103) = 0.26, *p* = 0.009, and not significant at 30 m, *r*(103) = 0.07, *p* = 0.515. Couple concordance for nonattuned mind related comments was not significant at 4 m, *r*(103) = 0.17, *p* = 0.083, and at 12 m, *r*(103) = −0.00, *p* = 0.994, but significant at 30 m, *r*(103) = 0.23, *p* = 0.019.

#### Temporal Continuity and Differences between Parents

IllingworthLastly, we tested the temporal continuity as well as differences between fathers’ and mothers’ use of appropriate and nonattuned mind-related comments across time. For appropriate mind-related comments, a significant effect was found for time, *F*(2, 206) = 61.45, *p* < 0.001, ƞ_p_^2^ = 0.37. Sidaks pairwise comparisons showed that parents produced significantly more appropriate mind-related comments at 4 m than at 12 m (*M*_4m_ = 7.74, *SE* = 0.39, *M*_12m_ = 5.32, *SE* = 0.26), *p* < 0.001, more at 12 m than at 30 m (*M*_30m_ = 3.88, *SE* = 0.16), *p* < 0.001), and more at 4 m than at 30 m, *p* < 0.001. Mothers and fathers did not differ in the amount of appropriate mind-related comments, *F*(1, 103) = 1.89, *p* = 0.172, ƞ_p_^2^ = 0.02. The interaction effect between parent gender and time was not significant, *F*(2, 206) = 0.04, *p* = 0.915, ƞ_p_^2^ = 0.00. To conclude, on average, fathers and mothers showed similar temporal patterns in their production of appropriate mind-related comments, with a significant decrease from 4 m to 30 m.

For parents’ nonattuned mind-related comments, a significant effect of time was found, *F*(2, 206) = 19.73, *p* < 0.001, ƞ_p_^2^ = 0.16. Parents produced significantly more nonattuned mind-related comments at 4 m than at 12 m (*M*_4m_ = 1.05, *SE* = 0.14, *M*_12m_ = 0.63, *SE* = 0.07), *p* = 0.006, at 12 m than at 30 m (*M*_30m_ = 0.30, *SE* = 0.04), *p* < 0.001, and at 4 m than at 30 m, *p* < 0.001. A significant effect of parent gender was also found, *F*(1, 103) = 18.69, *p* < 0.001, ƞ_p_^2^ = 0.16. On average, fathers produced more nonattuned mind-related comments than mothers (*M*_father_ = 0.90, *SE* = 0.11, *M*_mother_ = 0.42, *SE* = 0.04), *p* < 0.001. These results were qualified by a significant interaction effect between parent gender and time, *F*(2, 180) = 5.97, *p* = 0.003, ƞ_p_^2^ = 0.06. At the age of 4 m and 12 m fathers produced significantly more nonattuned mind-related comments than mothers, *p* = 0.002 and *p* < 0.001, respectively, while no significant difference was found between parents at 30 m, *p* = 0.230. Thus, a general decrease of nonattuned mind-related comments was found from 4 m to 30 m for both parents. On average, fathers produced more nonattuned comments than mothers at 4 m and 12 m, while no difference was found at 30 m.

### Prediction of Children’s Social Competence and Behavior Problems

#### Appropriate Mind-Mindedness

Table 2 summarizes the hierarchical regression analyses of the predictive values of fathers’ and mothers’ appropriate mind-mindedness on children’s social competence, externalizing and internalizing behaviors at 4.5 y. Parental appropriate mind-mindedness at 4 m, 12 m, and 30 m (step1, step 2 and step 3) accounted for nonsignificant proportions of the variance in children’s social competence and internalizing behavior. Concerning the prediction of children’s externalizing behavior, the model including appropriate mind-mindedness of both parents at 30 m was significant (step 3), explaining 17% of the total variation in externalizing behavior. Besides, a significant interaction was found between appropriate mind-related comments of mothers and fathers.

**Table 2:** Hierarchical Multiple Regressions for Parents’ Appropriate Mind-Mindedness at 4 m (step 1), 12 m (step 2) and 30 m (step 3) as predictors of Children’s Social Competence, Externalizing and Internalizing problems

	Social competence			Externalizing behavior			Internalizing behavior		
	Step1	Step2	Step 3	Step1	Step2	Step 3	Step1	Step2	Step 3
*R*^2^	0.03	0.04	0.10	0.05	0.07	0.17	0.05	0.08	0.12
*F*	0.87	0.69	1.10	1.91	1.18	2.08	1.74	1.39	1.46
*p*	0.459	0.655	0.373	0.133	0.324	0.039	0.163	0.228	0.176
*F* ∆*R*		0.53	1.87		0.48	3.68		1.03	1.54
*p F* ∆*R*		0.662	0.141		0.696	0.015		0.383	0.208
	Coefficients								
AMRC mother 4 m	−0.00	0.03	−0.02	−0.06	−0.07	0.00	0.05	0.09	0.09
AMRC father 4 m	0.14	0.11	0.09	−0.20	−0.16	−07	−0.03	−0.05	−0.06
AMRC mother x father 4 m	0.07	0.08	0.07	−0.10	−0.08	−0.06	−0.22*	−0.18	−0.20
AMRC mother 12 m		−0.11	−0.01		0.08	0.01		−0.13	−0.12
AMRC father 12 m		0.11	0.08		−0.11	−0.09		0.11	0.09
AMRC mother x father 12 m		0.01	0.03		−0.06	−08		−0.09	−0.02
AMRC mother 30 m			−15			−0.01			0.08
AMRC father 30 m			0.15			−14			0.20
AMRC mother x father 30 m			−0.13			0.29**			0.09

AMRC = Appropriate mind related comments; *p < 0.05; **p < 0.01

**Fig. 2:**
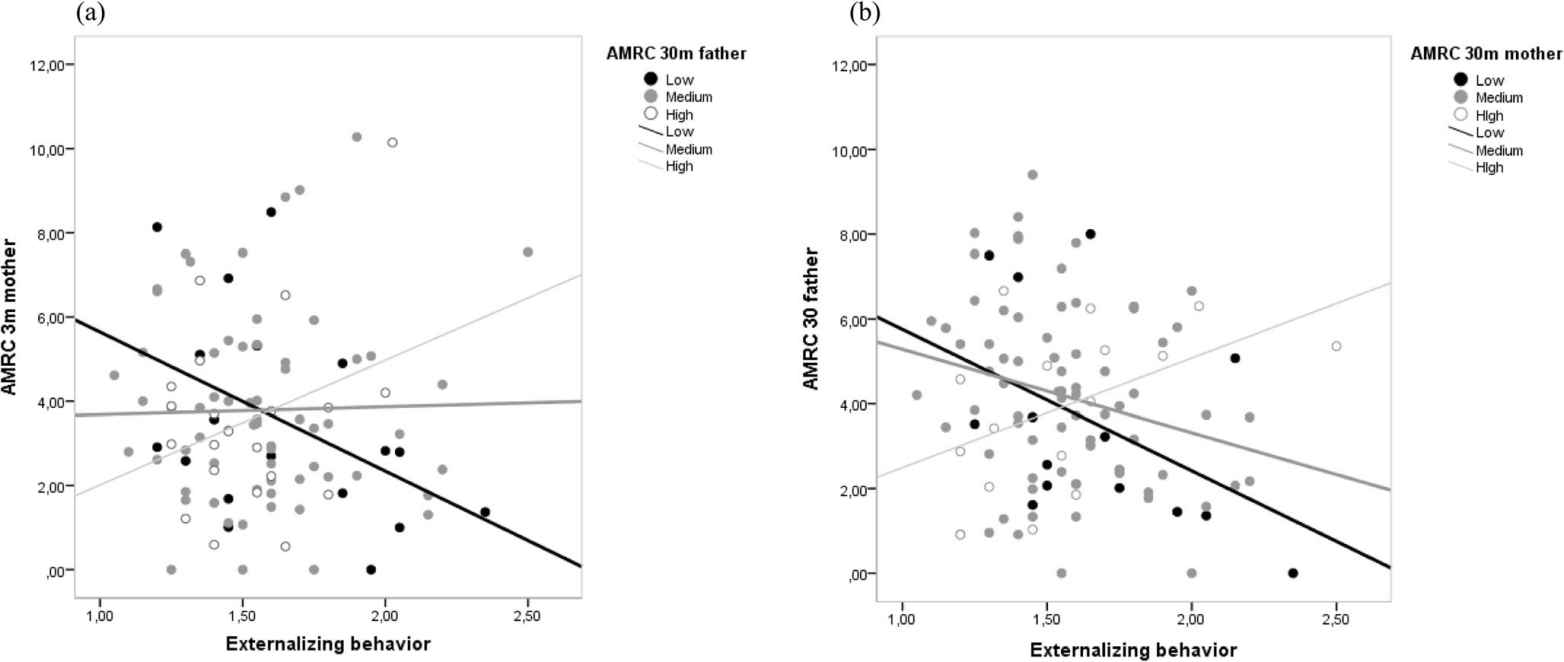
**a** Simple slopes plot of mothers appropriate mind-related comments at 30 m predicting children’s externalizing behavior for 1 *SD* below the mean (Low), the mean (Medium), and 1 *SD* above the mean (High) of fathers’ appropriate mind-related comments; **b** Simple slopes plot of fathers’ appropriate mind-related comments (AMRC) at 30 months predicting children’s externalizing behavior for 1 *SD* below the mean (Low), the mean (Medium), and 1 *SD* above the mean (High) of mothers’ appropriate mind-related comments. Note: AMRC: appropriate mind-related comments

The significance of this interaction effect was confirmed by the PROCESS moderation analysis in which we added mothers’ appropriate mind-related comments at 30 m as predictor of children’s externalizing behavior, fathers’ level of appropriate mind-mindedness (low, medium, high) at 30 m as moderator, and mothers’ and fathers’ (appropriate and nonattuned) mind-mindedness at 4 m and at 12 m and their interactions as covariates, B = 0.02 (*SE* < 0.01), *t*(94) = 3.02, *p* = 0.003, 95% CI [0.01, 0.03]. We probed the interaction with the pick-a-point approach (Fig. 2a). When fathers’ appropriate mind-mindedness was low, mothers’ use of appropriate mind-related comments was significantly negatively related to children’s externalizing behavior, B = −0.04 (*SE* = 0.02), *t*(100) = −2.53, *p* = 0.013, 95% CI [−0.07, −0.01]. When fathers’ appropriate mind-mindedness was medium or high, no significant association was found between mothers’ use of appropriate mind-related comments and children’s externalizing behavior, B = −0.00 (*SE* = 0.01), *t*(100) = −0.01, *p* = 0.989, 95% CI [−0.03, 0.03], and b = 0.04 (*SE* = 0.02), *t*(100) = −1.88, *p* = 0.064, 95% CI [−0.00, 0.08], respectively.

To further understand the interplay between mothers’ and fathers’ mind-mindedness, we again probed the same interaction with fathers’ use of appropriate mind-related comments as predictor of children’s externalizing behavior, and mothers’ level of appropriate mind-mindedness as moderator, and mothers’ and fathers’ (appropriate and nonattuned) mind-mindedness and their interactions at 4 m and at 12 m as covariates (Fig. 2b). When mothers’ appropriate mind-mindedness was low, a significant negative association was found between fathers’ use of appropriate mind-related comments at 30 m and children’s externalizing behavior, B = −0.06 (*SE* = 0.02), *t*(94) = −3.11, *p* = 0.002, 95% CI [−0.10, −0.02]. There was no significant association between fathers’ use of appropriate mind-related comments and children’s externalizing behavior when mothers’ appropriate mind-mindedness was medium, B = −0.02 (*SE* = 0.01), *t*(94) = −1.34, *p* = 0.182, 95% CI [−0.05, 0.01], or high, B = 0.02 (*SE* = 0.02), *t*(94) = 1.20, *p* = 0.235, 95% CI [−0.02, 0.06]. To summarize, children’s externalizing behavior at 4.5 y was predicted by low levels of appropriate mind-mindedness in both parents, but not by a low level of appropriate mind-mindedness in only one parent or high levels in both parents.

#### Nonattuned Mind-Mindedness

Results of the hierarchical regression analyses on the predictive values of fathers’ and mothers’ nonattuned mind-mindedness on children’s social competence and externalizing and internalizing problems are reported in Table 3. The model predicting children’s social competence was significant at step 2, explaining 13% of the variance. The standardized coefficients show that mothers’ and fathers’ nonattuned mind-mindedness at 12 m was significantly negatively related to children’s social competence at 4.5 y. The model remained significant at step 3, but parents’ mind-mindedness at 30 m did not have an effect above and beyond the effect of 12 m (∆*R* = 0.05). Also, the model predicting children’s externalizing behavior was significant at step 2, explaining 13% of the variance. Although this model was also significant at step 3, again, no significant additional variance was explained by parents’ mind-mindedness at 30 m (∆*R* = 0.04). In both models, mothers’ use of nonattuned mind-related comments at 12 m was a significant predictor of behavior problems. Additionally, at step 3, fathers’ use of nonattuned mind-related comments at 30 m became a significant predictor of externalizing problems. The model predicting children’s internalizing problems was not significant. To summarize, children’s social competence at 4.5 y was predicted by lower levels of nonattuned mind-mindedness in mothers and fathers at 12 m. Children’s externalizing behavior was predicted by high levels of nonattuned mind-mindedness in mothers at 12 m and 30 m and in fathers at 30 m. No significant association was found between children’s internalizing behavior and parents’ nonattuned mind-mindedness.

**Table 3:** Hierarchical Multiple Regressions for Parents’ Maternal Nonattuned Mind-Mindedness at 4 m (step 1), 12 m (step 2) and 30 m (step 3) as predictors of Children’s Social Competence, Externalizing and Internalizing problems

	Social competence			Externalizing behavior			Internalizing behavior		
	Step1	Step2	Step 3	Step1	Step2	Step 3	Step1	Step2	Step 3
*R*^2^	0.01	0.13	0.18	0.05	0.13	0.17	0.01	0.10	0.15
*F*	0.40	2.34	2.30	0.71	2.37	2.15	0.23	1.74	1.86
*p*	0.756	0.037	0.022	0.170	0.036	0.033	0.876	0.121	0.068
*F* ∆*R*		4.25	2.06		2.92	1.62		3.23	2.00
*p F* ∆*R*		0.007	0.111		0.038	0.190		0.026	0.119
	Coefficients								
NAMRC mother 4 m	−0.03	−0.05	−0.08	0.05	0.07	0.11	−0.02	0.00	−0.03
NAMRC father 4 m	−0.09	0.02	−0.02	0.17	0.13	0.13	−0.07	−0.06	−0.05
NAMRC mother x father 4 m	0.09	0.07	0.06	−0.18	−0.16	−0.14	−0.02	0.02	0.01
NAMRC mother 12 m		−0.24*	−0.26**		0.22*	0.25*		0.30**	0.31**
NAMRC father 12 m		−0.24*	−0.17		0.18	0.14		0.02	0.00
NAMRC mother x father 12 m		−0.08	−0.11		0.04	0.08		−0.04	−0.03
NAMRC mother 30 m			−0.07			−0.03			0.05
NAMRC father 30 m			−0.18			0.23*			−0.12
NAMRC mother x father 30 m			−0.08			−0.02			0.23*

NAMRC = Nonattuned mind-related comments; *p < 0.05; **p < 0.01

## Discussion

This prospective longitudinal study investigated: a) the stability and continuity of maternal and paternal mind-mindedness from infancy to toddlerhood; b) the concordance and differences between mothers and fathers in mind-mindedness, and c) whether parents’ mind-mindedness predicts children’s social competence and behavior problems at 4.5 y. Mothers’ appropriate mind-related comments showed moderate stability from 4 m to 12 m and from 12 m to 30 m, whereas their nonattuned comments were unstable over time. Fathers’ appropriate mind-related comments showed moderate stability from 4 m to 12 m but not from 12 m to 30 m, whereas their nonattuned comments showed strong stability from 4 m to 12 m and from 12 m to 30 m. Concerning the continuity, both parents’ use of appropriate and nonattuned mind-mindedness decreased across age. Concordance within couples was only found for appropriate mind-related comments at 12 m, and nonattuned mind-related comments at 30 m. We found no differences between parents in the quantity of production of appropriate mind-mindedness. Fathers, however, produced on average more nonattuned mind-related comments than mothers at 4 m and 12 m. These findings add to scant data about the stability and continuity of mind-mindedness (McMahon et al. 2016; Meins et al. 2011) and about differences and similarities between parents (Arnott and Meins 2007; Lundy 2003, 2013). Except for fathers’ higher levels of nonattuned mindedness than mothers in the first year, differences between parents seem more due to individual characteristics than to sex. These results suggest that parents expose the child to various forms of mind-related speech, affecting the child in unique and sometimes complementary ways.

When looking at the predictive value of mind-mindedness, mothers and fathers seem to compensate each other in preventing externalizing problems. That is, only when both parents showed low proportions of appropriate mind-related speech at 30 m, children had higher levels of externalizing problems at 4.5 y. Furthermore, less social competence was predicted by mothers’ and fathers’ nonattuned mind-related comments at 12 m, and externalizing problems were predicted by mothers’ nonattuned mind-mindedness at 12 m and by fathers’ nonattuned mind-mindedness at 30 m. No predictive value of parents’ mind-mindedness was found for internalizing problems. These results, their relevance, and their implications will be further discussed in the following sections.

### Temporal Stability, Parental Concordance, and Differences between Parents

The temporal stability of mothers for appropriate, but not for nonattuned, mind-mindedness confirms previous findings in the first two years of life (Kirk et al. 2015; McMahon et al. 2016; Meins et al. 2011), and add to previous studies by extending the stability of mind-mindedness until 30 m. In other words, mothers who explicitly demonstrate their understanding of the child’s mental states in the first year of life, also maintain this tendency during toddlerhood, after children start to speak. For fathers, the use of appropriate mind-related comments was found to be stable only during infancy, while their use of nonattuned mind-related comments remained stable till 30 m. Thus, when fathers tend to misread their infant’s thoughts and feelings in the first months, they appear more likely to continue misreading in the years ahead. Further, for both fathers and mothers, we found no direct temporal associations between mind-related comments from 4 m to 30 m (i.e., without the mediation of 12 m), suggesting a truly sequential nature of parents’ mind-mindedness from infancy to toddlerhood, and the possible influence of other factors such as shared parenting experiences, children’s language, and their socio-emotional development. These factors and their interactions may make the longterm temporal stability of parents’ mind-mindedness weaker. The results add to the recent growing body of research on the applicability of observation-based measures of mind-mindedness in infancy and beyond (Colonnesi et al. 2017; Fishburn 2017; Illingworth et al. 2016; Lundy 2013), and offer some evidence about the differences between fathers and mothers.

Concerning the temporal continuity, for both parents, the proportions of appropriate and nonattuned mind-mindedness show a temporal pattern of decrease from 4 m to 30 m, even though they remain positively associated with the previous assessments. This pattern may be the result of changes in the development of parent-child interactions. From the second year of life, infants become increasingly able to communicate in an intentional way (Carpendale and Lewis 2004), and before 30 m, the acquisition of language occurs. As children communicate their perspectives and mental states more explicitly, parents’ tendency to explicate their appreciation of the child’s mental states might decrease (Meins and Fernyhough 2015). Besides, when the child grows up parents’ language becomes more complex and repetitions of comments decrease (i.e., repetitions in observations with infants are counted as additional mind-related comments). Also, the interactional context changes from an almost exclusive face-to-face setting at 4 m to a more interactive and varied setting at 12 m and 30 m, in which the child becomes increasingly free to explore and the parent can alternate face-to-face episodes with moments of observation of the child’s exploration of the environment.

Concordance between parents was found only for appropriate mind-related comments at 12 m, and for nonattuned mind-related comments at 30 m, with small to medium effect sizes. The lack of concordance at 4 m might be explained by the impact of the possible antecedents of mind-mindedness, like parents’ own state of mind as a result of past attachment experiences (Arnott and Meins 2007; Bernier and Dozier 2003; Demers et al. 2010), individual mentalizing capacities (Sharp & Fonagy, 2008). Thus, at the beginning of parenthood, mind-mindedness could be seen primarily as a specific trait of a parent (Meins 1999; Meins et al. 2011). By the age of 12 m, parents have gotten to know their (first-born) infant better and have had more time to adopt similar modes of interacting with their infant and being attuned to the infant’s mental states (Arnott and Meins 2007; Deschênes et al. 2014; Lundy 2013). When the child is 30 m, couple’s concordance in the use of appropriate mind-mindedness might decrease because the relationship between the child and each parent has become more distinguished and unique. Concordance for nonattuned mind-mindedness, however, might increase as a result of characteristics of the child that have the same impact on the relationship with both parents (e.g., children with a difficult temperament or delayed development of socio-cognitive or language skills). These findings suggest that parents’ mind-mindedness is a unique characteristic intertwined with the development of parent-child relationships (Meins et al. 2014).

Fathers and mothers produced similar amounts of appropriate mind-related comments during all three assessment time-points, confirming previous results (Arnott and Meins 2007; Lundy 2003, 2013). In general, fathers and mothers seem to be similar in how often they are attuned to their child’s mental states. Fathers’ involvement in child-rearing has substantially increased in the last decades, reducing differences between parents in time spent with their child as well as in the quality of their relationship with the child (Cabrera et al. 2000). Fathers in the present study, however, did produce at 4 m and 12 m a higher number of nonattuned mind-related comments than mothers. This result is in line with previous findings on fathers’ mind-mindedness (Arnott and Meins 2007), and with the assumption that mothers, as early primary caregivers, generally preserve a more accurate understanding of their child’s inner states (Hallers-Haalboom et al. 2014).

### Predicting Social Competence and Behavior Problems

This study found that when both parents were low in appropriate mind-mindedness at 30 m, their child more often showed externalizing problems at 4.5 years. Moreover, the results show that when one parent is low but the other is average or high in appropriate mind-mind-mindedness, the lack of one parent’s appropriate mind-mindedness does not predict the child’s externalizing problems. The results suggest a joint effect of mothers’ and fathers’ appropriate mind-mind-mindedness on the development of children’s behavior problems. The results only partially align with Meins et al. (2013a, b) who reported that maternal mind-mindedness associated positively with externalizing behavior problems in low, but not in high, SES families. In the present study’s sample, consisting of primarily medium to high SES families, there was also no relation between a single parent’s mind-mindedness and children’s behavior problems. Possibly in high SES families there is less risk of accumulating factors that can impede the child’s development, and one parent might still compensate for the other parent’s lack of mind-mindedness, protecting the child from developing externalizing problems. Although these findings should be replicated in samples that are more heterogeneous in terms of SES, they indicate the importance of studying the joint effect of parents’ mind-mindedness on children’s socio-emotional development.

Proportions of nonattuned mind-related comments predicted lower social competence as well as more externalizing behavior at 4.5 y. These results indicate that mothers’, as well as fathers’, frequent misinterpretations of their child’s behavior and mind have a predictive value in the onset of child psychopathology. Nonattuned mind-related speech could lay a foundation for problems in social interactions and communication. Mind-mindedness offers, already from infancy, a meeting of minds between the parent and the infant, a way to share intentionality and emotions. Regular misinterpretation of the child’s mental states might hinder children in developing the capacity to understand their own mental states appropriately and to recognize the same mental states in others (Fonagy et al. 2004). In turn, children’s difficulty with comprehending their own and others’ mental states is associated with lower social competence and more behavior problems (Centifanti et al. 2016; Liddle and Nettle 2006).

The association between parents’ early nonattuned mind-mindedness and insecure attachment may also underlie the results mentioned above. For example, Meins et al. (2012) found that nonattuned, but not appropriate, mind-mindedness discriminates between insecure-avoidant, insecure-resistant, and insecure-disorganized child-mother relationships. Similarly, in a recent meta-analysis, nonattuned mind-mindedness predicted children’s insecure attachment more strongly than the absence of appropriate comments (*r* = 0.45 versus *r* = 0.26; Zeegers et al. 2017). Moreover, McMahon and Newey (2018) found that mothers’ use of nonattuned mind-related comments during a stressful situation (Still-Face Paradigm) was associated with dysregulated affect in their 6-month-old infants. Only when children are securely attached, they feel comfortable to explore their environment freely, engaging more easily in new social experiences. Further, only when children are securely attached, they are able to regulate their emotions adequately through the contact with the caregiver, which later turns into healthy self-regulation. Indeed, insecurely attached children are at higher risk for emotion regulation problems, as well as externalizing and internalizing behavior problems (Colonnesi et al. 2011; Fearon et al. 2010). A recent meta-analysis compared the levels of behavior problems in securely and insecurely attached children (Madigan et al. 2016), and reported medium effect sizes. Insecurely attached children presented more internalizing (*d* = 0.58) and externalizing problems (*d* =0. 49) than securely attached children. Insecure attachment may, therefore, be one of the mechanisms linking parental mind-mindedness to children’s behavior problems. Future research on the association between parents’ mind-mindedness and children’s difficulties should, therefore, include a measure of attachment, and should examine parents’ spontaneous use of mind-related comments in attachment-activating rather than free-play contexts, to increase the specificity and sensitivity of the measure (Bigelow et al. 2015; McMahon and Bernier 2017; Milligan et al. 2015; McMahon and Newey 2018).

A relevant issue is whether parents’ mind-mindedness predicts children’s social competence, internalizing, and externalizing problems over and above more established predictors, such as parental sensitivity, supportive behavior, or warmth (e.g., Besemer et al. 2016; Pinquart 2017a, 2017b). The assessment of mind-mindedness originated from research that attempted to refine the operationalization of parental sensitivity and therefore partially taps into the same concept (see Zeegers et al. 2017). For this reason, studies have focused mainly on comparing these two parenting features as predictors of child development. A meta-analytic study supported a model in which mind-mindedness uniquely predicted infant-parent attachment security, over and above parental sensitivity (Zeegers et al. 2017). Also, at 6 months, mothers’ use of non-attuned mind-related comments has been shown to correlate with infants’ negative affect, independent of maternal emotional availability (McMahon and Newey 2018); mothers’ appropriate mind-mindedness at 12 months has shown to predict children’s effort control and school readiness, independent of mothers’ sensitive behavior (Bernier et al. 2017); in low-SES families, mothers’ use of appropriate mind-related comments in the first year of life predicted fewer behavior problems at 61 months, again, independent of mothers’ sensitive behavior (Meins et al. 2013a, b). Lastly, a recent study found that mind-mindedness at 4 and 12 months predicted infants’ physiological emotion regulation over and above parenting quality (i.e., responsiveness, non-intrusiveness, warmth/affectivity, and positivity; Zeegers et al. 2018). Although the findings of Zeegers et al. (2018) suggest that the impact of parental mind-mindedness on child development might be independent of other parenting dimensions, more research is necessary to test the overlap between mind-mindedness and other parenting dimensions as a predictor of children’s social and behavior problems.

Although we did not find that parents’ use of mind-related comments predicted children’s internalizing behavior, we cannot exclude the presence of this association for two main reasons. First, children’s internalizing problems, such as anxiety and depression, generally show their onset in later childhood and during the transition to adolescence (Bongers et al. 2003). Second, children’s internalizing behaviors might be, because of their nature, less obvious and detectable than externalizing behaviors, increasing the possibility that emotional problems were underreported. Parent reports should, therefore, be combined with other assessments such as observational measures or teachers’ reports. The outcome that the regression model of nonattuned mind-mindedness predicting internalizing problems was nearly significant supports these notions, and points out that the relation between mind-mindedness and internalizing problems requires further study.

### Limitations

Our findings should be interpreted in light of some limitations. The parents who participated in this study were relatively highly educated. Therefore, the results have limited generalizability to a low- or middle-educated population. Another important limitation is that children’s social competence and behavior problems were assessed using parental reports. Although we averaged parents’ scores to obtain a more robust measure of child behavior, it could be that parents’ level of mind-mindedness influences their ability to evaluate their child’s behavior. Moreover, we did not test the possible moderation effects of children’s individual characteristics such as language, cognitive development, or temperament. These characteristics might impact how susceptible children are to parental behavior, including mind-mindedness (Pluess and Belsky 2009). Finally, parents’ use of nonattuned mind-related comments was infrequent: its effect should, therefore, be interpreted with caution.

## Conclusion

This study highlights the combined impact of fathers’ and mothers’ mind-mindedness on children’s early social competence and behavior problems. Our findings support the assumption that mind-mindedness is not a unique characteristic of the mother-infant relationship, but a characteristic of both parents-child relationship. Parental mind-mindedness seems to evolve from infancy to childhood, maintaining some stability but also changing, presumably because mind-mindedness is increasingly influenced by interactions among family members (i.e., parent-child, parent-parent). Along the same line, both mothers’ and fathers’ mind-mindedness in infancy and toddlerhood can be risk factors for psychopathology in early childhood.

## References

[CR1] Arnott B, Meins E (2007). Links among antenatal attachment representations, postnatal mind-mindedness, and infant attachment security: A preliminary study of mothers and fathers. Bulletin of the Menninger Clinic.

[CR2] Benjamini Y, Hochberg Y (1995). Controlling the false discovery rate: A practical and powerful approach to multiple testing. Journal of the Royal statistical society: series B (Methodological).

[CR3] Bernier A, Dozier M (2003). Bridging the attachment transmission gap: The role of maternal mind-mindedness. International Journal of Behavioral Development.

[CR4] Bernier A, McMahon CA, Perrier R (2017). Maternal mind-mindedness and children’s school readiness: A longitudinal study of developmental processes. Developmental Psychology.

[CR5] Besemer S, Loeber R, Hinshaw SP, Pardini DA (2016). Bidirectional associations between externalizing behavior problems and maladaptive parenting within parent-son dyads across childhood. Journal of Abnormal Child Psychology.

[CR6] Bigelow AE, Power M, Bulmer M, Gerrior K (2015). The relation between mothers' mirroring of infants' behavior and maternal mind-mindedness. Infancy.

[CR7] Blair BL, Perry NB, O'brien M, Calkins SD, Keane SP, Shanahan L (2015). Identifying developmental cascades among differentiated dimensions of social competence and emotion regulation. Developmental Psychology.

[CR8] Bollen KA, Curran PJ (2004). Autoregressive latent trajectory (ALT) models a synthesis of two traditions. Sociological Methods & Research.

[CR9] Bongers IL, Koot HM, Van der Ende J, Verhulst FC (2003). The normative development of child and adolescent problem behavior. Journal of Abnormal Psychology.

[CR10] Cabrera NJ, Tamis-LeMonda CS, Bradley RH, Hoffert S, Lamb ME (2000). Fatherhood in the twenty-first century. Child Development.

[CR11] Camisasca E, Miragoli S, Ionio C, Milani L, Di Blasio P (2018). Post-partum depressive symptoms and child behavior: The mediational role of maternal mind-mindedness. Children's Health Care.

[CR12] Carpendale JIM, Lewis C (2004). Constructing an understanding of mind: The development of children's social understanding within social interaction. Behavioral and Brain Sciences.

[CR13] Centifanti L, Meins E, Fernyhough C (2016). Callous-unemotional traits and impulsivity: Distinct longitudinal relations with mind-mindedness and understanding of others. Journal of Child Psychology and Psychiatry.

[CR14] Colonnesi C, Draijer EM, Stams JJMG, Van der Bruggen CO, Bögels SM, Noom MJ (2011). The relation between insecure attachment and child anxiety: A meta-analytic review. Journal of Clinical Child & Adolescent Psychology.

[CR15] Colonnesi C, van Polanen M, Tavecchio LW, Fukkink RG (2017). Mind-mindedness of male and female caregivers in childcare and the relation to sensitivity and attachment: An exploratory study. Infant Behavior and Development.

[CR16] Crucianelli L, Wheatley L, Filippetti ML, Jenkinson PM, Kirk E, Fotopoulou AK (2018). The mindedness of maternal touch: An investigation of maternal mind-mindedness and mother-infant touch interactions. Developmental Cognitive Neuroscience.

[CR17] Demers I, Bernier A, Tarabulsy GM, Provost MA (2010). Maternal and child characteristics as antecedents of maternal mind-mindedness. Infant Mental Health Journal.

[CR18] Deschênes M, Bernier A, Jarry-Boileau V, St-Laurent D (2014). Concordance between the quality of maternal and paternal parenting behavior within couples. The Journal of Genetic Psychology.

[CR19] de Vente, W., Majdandžić, M., Colonnesi, C., & Bögels, S. M. (2011). Intergenerational transmission of social anxiety: The role of paternal and maternal fear of negative child evaluation and parenting behaviour. *Journal of Experimental Psychopathology*, *2*, 509–530.

[CR20] Devine RT, White N, Ensor R, Hughes C (2016). Theory of mind in middle childhood: Longitudinal associations with executive function and social competence. Developmental Psychology.

[CR21] Easterbrooks MA, Crossman MK, Caruso A, Raskin M, Miranda-Julian C (2017). Maternal mind–mindedness and toddler behavior problems: The moderating role of maternal trauma and posttraumatic stress. Development and Psychopathology.

[CR22] Fearon RP, Bakermans-Kranenburg MJ, Van IJzendoorn MH, Lapsley AM, Roisman GI (2010). The significance of insecure attachment and disorganization in the development of children’s externalizing behavior: A meta-analytic study. Child Development.

[CR23] Fishburn, S. (2017). ‘*Thinking about Parenting’–The Role of Mind-Mindedness and Parental Cognitions in Parental Behaviour and Child Developmental Outcomes* (Doctoral dissertation, University of York).

[CR24] Fonagy P, Gergely G, Jurist E, Target M (2004). Affect regulation, mentalization, and the development of the self.

[CR25] Graham JW (2009). Missing data analysis: Making it work in the real world. Annual Review of Psychology.

[CR26] Groh AM, Fearon RP, Bakermans-Kranenburg MJ, Van IJzendoorn MH, Steele RD, Roisman GI (2014). The significance of attachment security for children’s social competence with peers: A meta-analytic study. Attachment & Human Development.

[CR27] Hallers-Haalboom ET, Mesman J, Groeneveld MG, Endendijk JJ, van Berkel SR, van der Pol LD, Bakermans-Kranenburg MJ (2014). Mothers, fathers, sons and daughters: Parental sensitivity in families with two children. Journal of Family Psychology.

[CR28] Hayes AF (2013). Introduction to mediation, moderation, and conditional process analysis: A regression-based approach.

[CR29] Illingworth G, MacLean M, Wiggs L (2016). Maternal mind-mindedness: Stability over time and consistency across relationships. European Journal of Developmental Psychology.

[CR30] Kirk E, Pine K, Wheatley L, Howlett N, Schulz J, Fletcher BC (2015). A longitudinal investigation of the relationship between maternal mind-mindedness and theory of mind. British Journal of Developmental Psychology.

[CR31] Kotler JC, McMahon RJ (2002). Differentiating anxious, aggressive, and socially competent preschool children: Validation of the social competence and behavior Evaluation-30 (parent version). Behaviour Research and Therapy.

[CR32] LaFreniere PJ, Dumas JE (1996). Social competence and behavior evaluation in children ages 3 to 6 years: The short form (SCBE-30). Psychological Assessment.

[CR33] Lamb ME, Lewis C, Lamb ME (2010). The development and significance of father–child relationships in two-parent families. The role of the father in child development.

[CR34] Laranjo J, Bernier A, Meins E, Carlson SM (2014). The roles of maternal mind-mindedness and infant security of attachment in predicting preschoolers’ understanding of visual perspective taking and false belief. Journal of Experimental Child Psychology.

[CR35] Liddle B, Nettle D (2006). Higher-order theory of mind and social competence in school-age children. Journal of Cultural and Evolutionary Psychology.

[CR36] Luman M, Van Meel CS, Oosterlaan J, Sergeant JA, Geurts HM (2009). Does reward frequency or magnitude drive reinforcement-learning in attention-deficit/hyperactivity disorder?. Psychiatry Research.

[CR37] Lundy BL (2003). Father-and mother-infant face-to-face interactions: Differences in mind-related comments and infant attachment?. Infant Behavior and Development.

[CR38] Lundy BL (2013). Paternal and maternal mind-mindedness and preschoolers' theory of mind: The mediating role of interactional attunement. Social Development.

[CR39] Luteijn EF, Serra M, Jackson S, Steenhuis MP, Althaus M, Volkmar F, Minderaa R (2000). How unspecified are disorders of children with a pervasive developmental disorder not otherwise specified? A study of social problems in children with PDD-NOS and ADHD. European Child & Adolescent Psychiatry.

[CR40] Luteijn EF, Minderaa RB, Jackson AE (2002). *VISK Handleiding. Vragenlijst voor Inventarisatie van Sociaal gedrag van Kinderen*.

[CR41] Madigan S, Brumariu LE, Villani V, Atkinson L, Lyons-Ruth K (2016). Representational and questionnaire measures of attachment: A meta-analysis of relations to child internalizing and externalizing problems. Psychological Bulletin.

[CR42] McMahon CA, Bernier A (2017). Twenty years of research on parental mind-mindedness: Empirical findings, theoretical and methodological challenges, and new directions. Developmental Review.

[CR43] McMahon C, Newey B (2018). Non-attuned mind-mindedness, infant negative affect, and emotional availability: Assessing mind-mindedness during the still-face paradigm. Infancy.

[CR44] McMahon C, Camberis AL, Berry S, Gibson F (2016). Maternal mind-mindedness: Relations with maternal–fetal attachment and stability in the first two years of life: Findings from an Australian prospective study. Infant Mental Health Journal.

[CR45] Meins E (1999). Sensitivity, security and internal working models: Bridging the transmission gap. Attachment & Human Development.

[CR46] Meins E (2013). Sensitive attunement to infants’ internal states: Operationalizing the construct of mind-mindedness. Attachment & Human Development.

[CR47] Meins, E., & Fernyhough, C. (2015). *Mind-mindedness coding manual, Version 2.2.* Unpublished manuscript. Durham University, Durham.

[CR48] Meins E, Fernyhough C, Wainwright R, Clark-Carter D, Das Gupta M, Fradly E, Tukey M (2003). Pathways to understanding mind: Construct validity and predictive validity of maternal mind-mindedness. Child Development.

[CR49] Meins E, Fernyhough C, Arnott B, Turner M, Leekam SR (2011). Mother- versus infant-centered correlates of maternal mind-mindedness in the first year of life. Infancy.

[CR50] Meins E, Fernyhough C, de Rosnay M, Arnott B, Leekam SR, Turner M (2012). Mind-mindedness as a multidimensional construct: Appropriate and nonattuned mind-related comments independently predict infant-mother attachment in a socially diverse sample. Infancy.

[CR51] Meins E, Centifanti LCM, Fernyhough C, Fishburn S (2013). Maternal mind-mindedness and children’s behavioral difficulties: Mitigating the impact of low socioeconomic status. Journal of Abnormal Child Psychology.

[CR52] Meins E, Fernyhough C, Arnott B, Leekam SR, de Rosnay M (2013). Mind-mindedness and theory of mind: Mediating roles of language and perspectival symbolic play. Child Development.

[CR53] Meins E, Fernyhough C, Harris-Waller J (2014). Is mind-mindedness trait-like or a quality of close relationships? Evidence from descriptions of significant others, famous people, and works of art. Cognition.

[CR54] Milligan K, Khoury JE, Benoit D, Atkinson L (2015). Maternal attachment and mind-mindedness: The role of emotional specificity. Attachment & Human Development.

[CR55] Möller, E. L., Majdandžić, M., de Vente, W., & Bögels, S. M. (2013). The evolutionary basis of sex differences in parenting and its relationship with child anxiety in Western societies. *Journal of Experimental Psychopathology, 4*, 88-117.

[CR56] Pinquart M (2017). Associations of parenting dimensions and styles with externalizing problems of children and adolescents: An updated meta-analysis. Developmental Psychology.

[CR57] Pinquart M (2017). Associations of parenting dimensions and styles with internalizing symptoms in children and adolescents: A meta-analysis. Marriage & Family Review.

[CR58] Pluess M, Belsky J (2009). Differential susceptibility to rearing experience: The case of childcare. Journal of Child Psychology & Psychiatry.

[CR59] Sharp, C., & Fonagy, P. (2008). The parent's capacity to treat the child as a psychological agent: Constructs, measures and implications for developmental psychopathology. *Social Development, 17*, 737-754.

[CR60] Tabachnick BG, Fidell LS (2013). Using multivariate statistics.

[CR61] Zeegers M, Colonnesi C, Stams GJ, Meins E (2017). Mind matters: A meta-analysis on parental Mentalization and sensitivity as predictors of infant-parent attachment. Psychological Bulletin.

[CR62] Zeegers MA, de Vente W, Nikolić M, Majdandžić M, Bögels SM, Colonnesi C (2018). Mothers’ and fathers’ mind-mindedness influences physiological emotion regulation of infants across the first year of life. Developmental Science.

