# Correction: Fathers’ and Mothers’ Early Mind-Mindedness Predicts Social Competence and Behavior Problems in Childhood

**DOI:** 10.1007/s10802-023-01036-1

**Published:** 2023-03-03

**Authors:** Cristina Colonnesi, Moniek A. J. Zeegers, Mirjana Majdandžić, Francisca J. A. van Steensel, Susan M. Bögels

**Affiliations:** 1 Research Institute of Child Development and Education, University of Amsterdam, Nieuwe Achtergracht 127, 1001 NG Amsterdam, The Netherlands; 2 Research Priority Area Yield, University of Amsterdam, Nieuwe Achtergracht 127, 1001 NG Amsterdam, The Netherlands

**Correction: Journal of Abnormal Child Psychology (2019) 47:1421–1435**

10.1007/s10802-019-00537-2

The original version of this article unfortunately contained two critical mistakes in our article.

In the **Abstract**:

" Hierarchical multiple regression analyses showed that, at 12 months, infrequent use of appropriate mind-related comments of both parents predicted children’s externalizing problems …." 12 months should be **30 months**.

And, in the **Discussion** (page 1432):

" This study found that when both parents were low in appropriate mind-mindedness at 12 m, their child more often showed externalizing problems at 4.5 years ", 12 months should be **30 months**.

The original article has been corrected.

